# Idiopathic macular telangiectasia type 1: Clinical, multimodal imaging features and response to treatment

**DOI:** 10.1007/s00417-025-07042-x

**Published:** 2025-12-20

**Authors:** Adriano Carnevali, Massimiliano Borselli, Pietro Bianchi, Giovanna Carnovale-Scalzo, Andrea Lucisano, Mariangela Romeo, Sabrina Vaccaro, Alessandra Mancini, Domenico Chisari, Raffaella Gioia, Vincenzo Mollace, Davide Allegrini, Mario R. Romano, Vincenzo Scorcia

**Affiliations:** 1 Ophthalmology Unit, Department of Surgical and Medical Sciences, University Magna Graecia, A.O.U.“Renato Dulbecco”, Catanzaro, 88100 Italy; 2 Department of Health Sciences, Institute of Research for Food Safety and Health (IRC-FSH), University Magna Graecia of Catanzaro, Catanzaro, 88100 Italy; 3 Eye Unit, Department of Medical and Surgical Specialties, Radiological Sciences, and Public Health, University of Brescia, Brescia, Italy; 4 Department of Ophthalmology, Humanitas Gavazzeni-Castelli, Bergamo, Italy; 5 Department of Biomedical Sciences, Humanitas University, Via Rita Levi Moltalcini 4, Pieve Emanuele-Milano, 20072 Italy

**Keywords:** Idiopathic macular telangiectasia, Intravitreal dexamethasone implant, Intravitreal Anti-VEGF therapy, MactTel type 1, Optical coherence tomography, Fluorescein angiography

## Abstract

**Purpose:**

To describe the clinical characteristics, multimodal imaging findings, and treatment response in patients with idiopathic macular telangiectasia type 1 (MactTel Type 1).

**Methods:**

This retrospective cohort study involved patients affected by idiopathic MactTel Type 1. Multimodal imaging with fundus photography, optical coherence tomography (OCT), and optical coherence tomography angiography (OCT-A) and fluorescein angiography were reviewed. Treatment response to intravitreal anti-vascular endothelial growth factor (anti-VEGF) and dexamethasone (DEX) implants was evaluated.

**Results:**

A retrospective, observational study including 10 eyes from 10 patients diagnosed with MactTel Type 1. All eyes exhibited telangiectasias and microaneurysms in the foveal region. OCT showed intraretinal cysts and macular thickening. OCT-A demonstrated a mild reduction in superficial capillary density and numerous telangiectasias in the deep capillary plexus, correlating with macular edema. Fluorescein angiography revealed prominent parafoveal capillaries with late-phase leakage. Moreover, the presence of equatorial leakage was identified in 20% of cases and vascular hyperfluorescence in 60% of cases. Complete response to treatment was observed in 40% patients.

**Conclusion:**

MactTel type 1 predominantly affects the temporal retinal quadrants. While anti-VEGF therapy is minimally effective, intravitreal DEX appears to be a promising alternative.

## Key Messages

*****What is known***:**

- MacTel Type 1 predominantly affects the inferotemporal macular quadrant, presenting with characteristic vascular alterations such as telangiectasias and microaneurysms.
- Anti-VEGF therapy has shown limited efficacy, as MacTel Type 1 is a non-neovascular condition; conversely, intravitreal dexamethasone (DEX) implants have demonstrated better outcomes by reducing macular edema and preserving anatomical integrity.

*****What is new***:**

- This study provides a detailed characterization of idiopathic MacTel Type 1, highlighting distinctive vascular changes—particularly in the inferotemporal retina—through multimodal imaging.
- It is confirmed that intravitreal DEX implants are a more effective therapeutic option than anti-VEGF agents, offering anatomical and functional outcomes with a mean follow-up of 3 years.

## Introduction

**Fig. 1:**
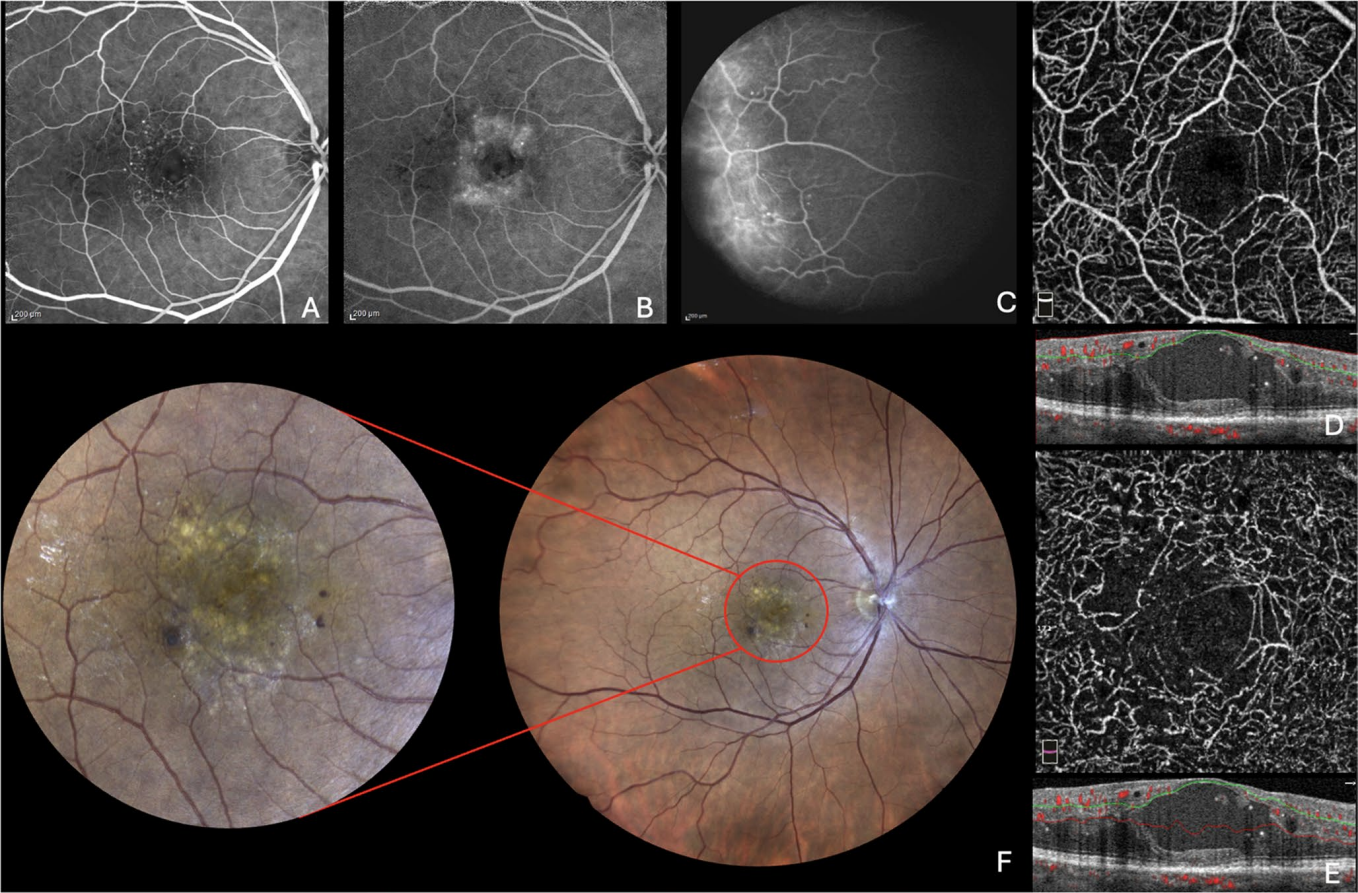
Figures **A**, and **B **represent the early and late phases of fluorescein angiography. Figure **C **represents the leakage sign in the temporal quadrant. Figures **D **and **G **show the superficial and deep capillary plexus with the respectively OCT slabs. The figure **E **represents the posterior pole on fundus color photography, with the red circle and red lines indicating a focus zoomed in on the macular area

**Fig. 2:**
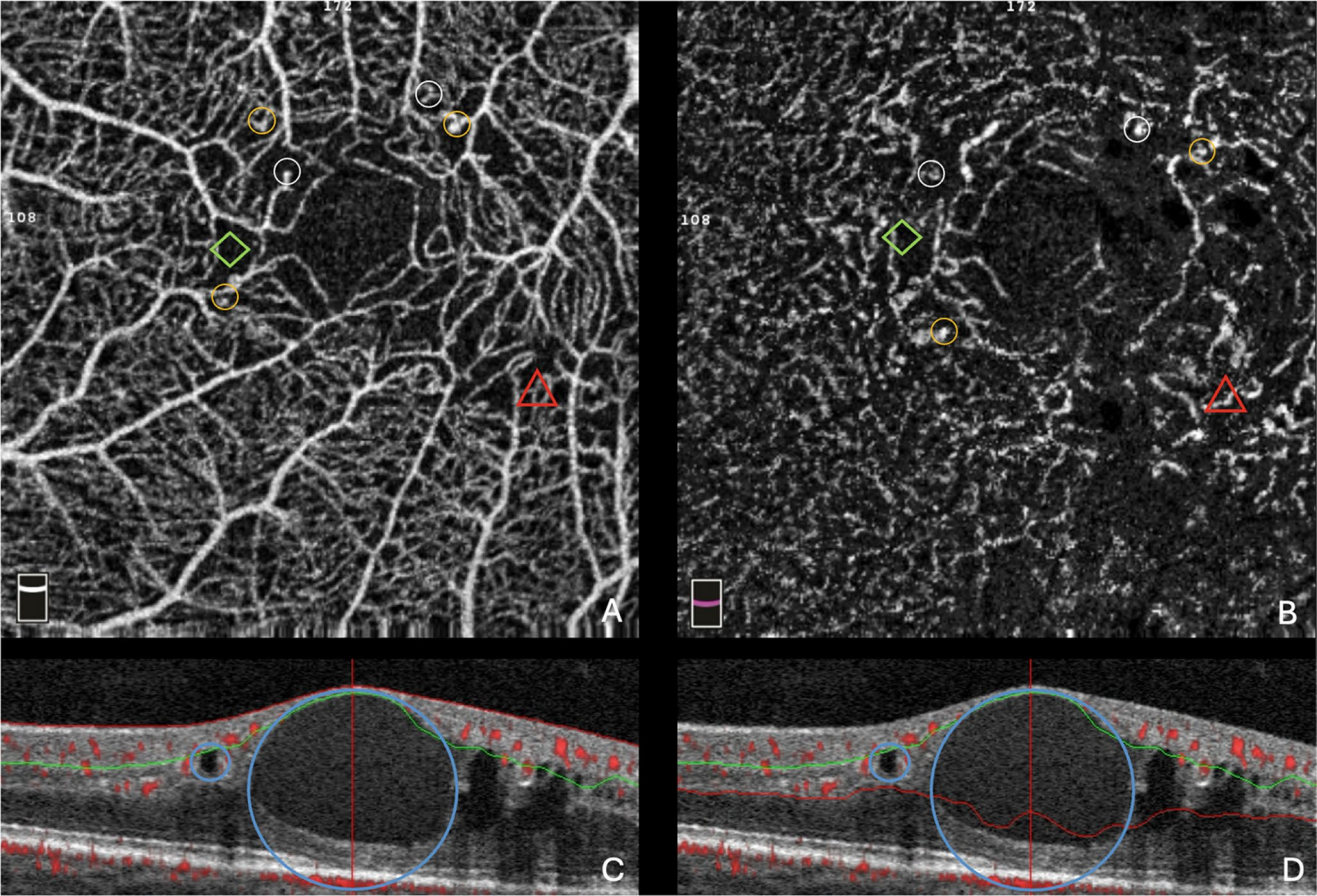
In Figure **A **and **B**, yellow circles indicate microaneurysms, white circles indicate telangiectasias, green rhombuses indicate ischemia, while the red triangles indicate vessel tortuosity. In Figure **C **and **D**, the blue circles indicate intraretinal cysts due to intraretinal fluid

Idiopathic macular telangiectasia type 1 (MactTel Type 1) is a rare, unilateral, sight threatening condition, typically affecting males aged 40 and 70 [1, 2]. This pathology is characterized by the presence of macular telangiectasias and cystoid macular edema, together with saccular dilatation, microaneurysms and lipid deposition mainly found in the temporal quadrant [3–5]. Some authors suggest that MactTel Type 1 may be considered a central variant of Coats Disease, which instead occurs in the periphery [6, 7]. The main cause for visual loss in these patients is macular edema caused by vascular exudation. Nevertheless, the level of exudation, cystoid macular edema, and subsequent visual acuity (VA) decline vary, as some patients may maintain satisfactory visual acuity for several years without any intervention. Vascular malformations may stay functionally normal for long periods before becoming pathologically active, and they may potentially resolve spontaneously [8]. Multimodal imaging covers a pivotal role for the evaluation of this pathology [9]. Fluorescein angiography (FA) reveals early-phase parafoveal microaneurysms, with late-phase leakage [10]. Telangiectatic vessels characterized by a rapid filling are clearly visible in both the superficial and deep juxtafoveolar capillary plexus [2, 10]. Capillary nonperfusion and retinal ischemia are occasionally noted in FA. Optical coherence tomography (OCT) is paramount to confirm central cystic or noncystic macular edema seen in FA, showing increased retinal thickness associated to intraretinal cysts and fluid accumulation at the macular level [11]. OCT may also reveal large intraretinal blood vessels impacting the outer nuclear layer and the external limiting membrane, potentially separating the outer nuclear from the outer plexiform layers [12]. Simultaneously, optical coherence tomography angiography (OCT-A) allows more comprehensive observation of retinal and choroidal vessels, detecting slight capillaries density reductions in the superficial foveal layer, various telangiectasias and dilated vessels in the deep capillary plexus, associated with macular edema and exudates (Figs. 1 and 2) [5].

Macular edema, as the main cause of morbidity, has prompted several treatments strategies, producing variable outcomes [13, 14]. Laser photocoagulation targeting ischemic areas to manage exudation and leaky capillary dilation, may lead to retinal scarring affecting prognosis [15, 16]. Conversely, intravitreal corticosteroids (e.g.; dexamethasone (DEX), and triamcinolone) have demonstrated good efficacy in managing edema. Anti-VEGF injections offer another approach, often used in combination with therapies like Ranibizumab or DEX alongiside laser photocoagulation [6]. Also, photocoagulation with verteporfin serves as another therapeutic option, although its effectiveness is minimally supported by scientific evidence.

This study aims to describe the clinical and multimodal imaging characteristics of MacTel Type 1, to elucidate its anatomical features, and to explore potential treatment responses through a long-term observational follow-up.

## Methods

From the institutional database comprising 26,312 patient records collected between 2013 and 2023 at the University of Magna Grecia in Catanzaro, Italy, an initial automated search was performed to identify cases labeled or clinically diagnosed as idiopathic MacTel Type 1. The study was conducted following the Declaration of Helsinki for research involving human subjects. All patients provided written informed consent.

In the first phase, automated filtering algorithms were applied to exclude patients presenting any systemic or ocular conditions listed in the exclusion criteria — such as diabetes mellitus, uncontrolled hypertension, major cardiovascular disease, diabetic or hypertensive retinopathy, retinal vein or artery occlusion, posterior uveitis, Coats disease with massive exudation, and significant media opacities. This step substantially reduced the initial dataset by removing all records associated with confounding vascular or inflammatory retinal disorders.

In the second phase, the remaining cases were individually reviewed by two ophthalmologists, who independently reviewed each patient’s multimodal imaging (OCT, FA, OCT-A, and fundus photography) and clinical chart to confirm the diagnosis of idiopathic MacTel Type 1 and to ensure adherence to the inclusion criteria (age > 18 years, predominantly unilateral telangiectasia with variably sized aneurysms, and absence of systemic or ocular confounders). For each patient, the first comprehensive ophthalmologic evaluation was assessed. Multimodal retinal imaging was reviewed, including FrfoA (*Spectralis HRA*,* Heidelberg Engineering*,* Germany*), fundus autofluorescence (*Spectralis HRA*,* Heidelberg Engineering*,* Germany*), spectral domain OCT (*RTVue OCT Optovue Inc.*,* Fremont*,* CA*,* USA*), OCT-A (*XR Avanti AngioVue*,* Optovue*,* Fremont*,* CA*,* USA or Zeiss Clarus 500 HD-OCT*,* Zeiss Meditec. Inc*,* Germany*) and color fundus photography (*EIDON AF*,* Centervue Padova*,* Italy*). An extensive analysis of records was conducted, where demographic informations (age, gender) were compiled. Clinical informations were gathered and summarized, including visual acuity, medical history (both ocular and systemic) and ocular treatments received. A qualitative analysis of OCT-A scans (3 mm × 3 mm centered at the fovea) was evaluated. The software automatically segmented scans into superficial (SCP) and the deep capillary plexus (DCP) slabs. The quantitative evaluations of SCP and DCP were conducted using the automated software algorithm of the AngioPlex, utilizing its default settings. The automatic segmentation provided by the OCT-A software was manually adjusted by two ophthalmologists (PB and MB) for accurate capillary, outer retinal layers, and choriocapillaris visualization. In case of persistent disagreement between the two ophthalmologists, a senior ophthalmologist and retina specialist (AC) was consulted to make the final adjudication. This facilitated the identification and analysis of teleangectasias, microaneurysms, ischemia, intraretinal cysts, and modifications in the foveal avascular zone (FAZ) both in the SCP and in the DCP. Assessments included OCT-A images and structural OCT B-scans along with FA analyzing the macular region into four quadrants (superior, S; nasal, N; inferior, I; and temporal, T). According to FA, early and late-phase macular photographs were evaluated along with the equatorial fields, which were further divided into four regions: equatorial superior (eS), equatorial nasal (eN), equatorial inferior (eI), and equatorial temporal (eT). Macular telangiectasias were identified as dilations at the extremities of macular capillary vessels and as hyperreflective spots on the En Face OCT-A, correlating with blood flow, while on showed them as hyperfluorescent areas. Microaneurysms appeared as non-extremity dilations of blood vessels and hyperreflective spots on the En Face OCT-A; corresponding to blood flow areas and showed hyperfluorescence on FA and indocyanine green angiography (ICGA). Vessel tortuosity, indicating a deviation from normal courses, was analyzed on En Face OCT-A and FA. Ischemia was detected as non-perfused areas on En Face OCT-A and FA. Intraretinal cysts were identified as organized fluid accumulations within the intraretinal layers on OCT-A. The FAZ was qualitatively evaluated on En Face OCT-A images. Any discernible alteration from its normal, circular-to-ovoid contour was considered irregular. This specifically included the presence of one or more of the following features: (i) focal indentations or scalloping of the border, (ii) marked asymmetry, or (iii) a clearly lobulated or angulated appearance. A diagnosis of MacTel type 1 was made in patients who fulfilled the inclusion criteria and demonstrated unilateral macular telangiectasia on multimodal imaging. Treatment responses to Anti-VEGF or DEX implant were categorized into (i) Complete response (resolution), defined as total clearance of fluid, including both intraretinal fluid (IRF), subretinal fluid (SRF); (ii) Partial response, characterized by a reduction of 25% from the initial central retinal thickness (CRT), accompanied by the persistence or newly emergence of IRF and/or SRF; (iii) Absent response, indicated by an elevation of CRT and related fluid levels, specifically IRF, SRF, compared to the initial measurements.

## Results

### Demographic and treatment analysis

**Fig. 3:**
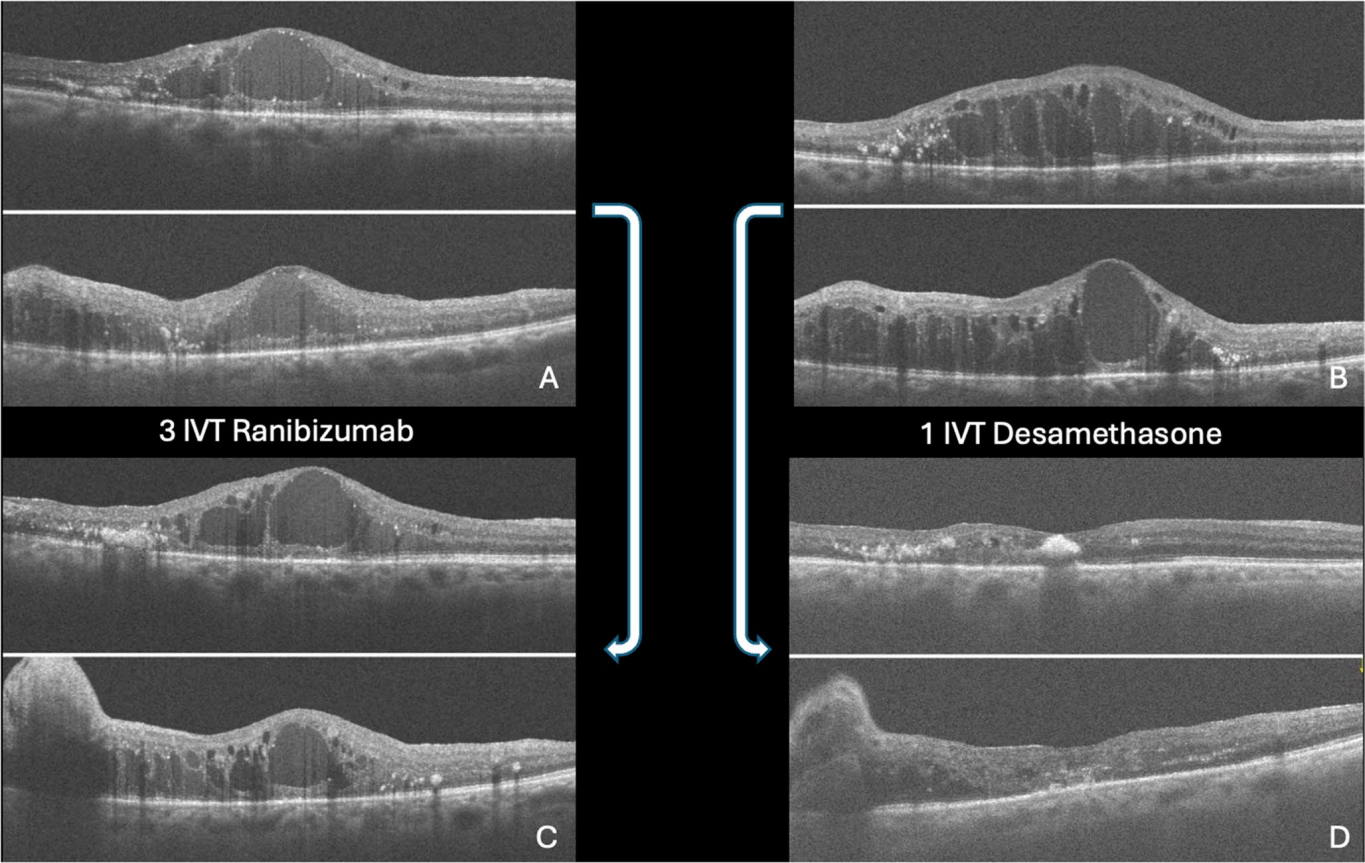
The figures **A **and **C **show a partial response to a cycle of Ranibizumab (each cycle is characterized by three Ranibizumab injections). Figures **B **and **D **show the total response to a single DEX injection

Overall, 26,302 patients were excluded as they did not meet all the necessary requirements for inclusion in the final analysis. Ten eyes from 10 patients (5 women and 5 men), all unilateral cases, with a mean age of the cohort was 42.22 ± 5.78 years (range 30.6 − 53.8 years; median 42 years), and the mean follow-up duration was 36.8 months (range +/- 41.5 months; median 39 months). At presentation, the mean BCVA was 30 letters in ETDRS. Six patients (60%) were treated with intravitreal injections of anti-VEGF and the mean number of injections was 3 (range 1–6; median 3 injections). Six eyes were previously treated with Anti-VEGF and subsequently with DEX, while 3 eyes were treated with DEX as first treatment. The total number of injections was 70: 36 Ranibizumab (46%), 15 Aflibercept (23%), and 19 DEX (29%). Initial treatments involved 6 Ranibizumab, 1 Aflibercept, and 3 DEX injections. Each cycle was considered as 3 injections of Anti-VEGF, the data showed 12 cycles of injections for Ranibizumab and 5 cycles of injections for Aflibercept. Outcomes varied, with 2 patients showing partial responses and 4 showing no response to Ranibizumab. For Aflibercept, 1 patient exhibited no response. Six patients were switched to DEX after Anti-VEGF treatments. Among those transitioning from Ranibizumab to DEX, 1 showed no response, 4 had partial responses, and 1 achieved a complete response (Fig. 3). Considering the Aflibercept to DEX switch, 1 patient exhibited a partial response. When DEX was used as the initial treatment, 3 patients displayed complete responses. Additionally, 2 patients initially received combined therapy, involving Anti-VEGF and Argon laser treatments in order to manage temporal leakage, feature observed in 70% of the patients. (Table 1)

**Table 1:** Overview of intravitreal injection treatments administered to the study patients

Therapy	*n*° of injections	*n*° of cycles of injections (1cycle = 3 injections)	Switch to DEX (*n*°patients)
Ranibizumab	36 (51,43%)	12	5
Aflibercept	15 (21,43%)	5	1
Dexamethasone	19 (27,14%)	-	-
TOT	70	17	6

*DEX* Dexamethasone

### Multimodal imaging analysis

All OCT-A qualitative analysis were reviewed. The SCP analysis showed revealed telangiectasia and microaneurysms as follows: S quadrant in 6 eyes (60%), N quadrant in 6 eyes (60%), I quadrant in 7 patients (70%), and T quadrant in 8 patients (80%). Vessels tortuosity in SCP was observed in 5 patients (50%) in the S, 3 patients (30%) in the N, 7 patients (70%) in the I, and 8 patients (80%) in the T quadrant. Retinal ischemia was observed in 6 patients (60%) in the S, 5 patients (50%) in the N, 8 patients (80%) in the I, and 7 eyes (70%) in the T quadrant. Intraretinal cysts were found in 4 eyes (40%) in the S, 3 eyes (30%) in the N, 2 patients (20%) in the I, and 4 patients (40%) in the T quadrant. Irregularity of the FAZ was noted in 3 patients (30%).

**Fig. 4:**
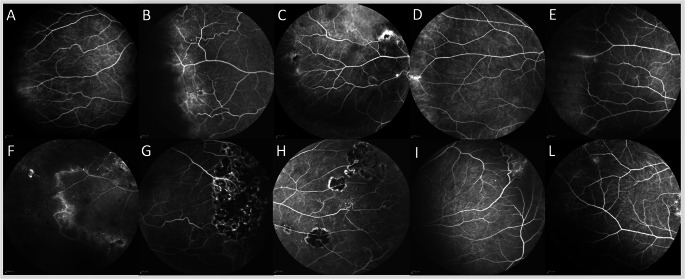
** A**–**F** Vascular hyperfluorescence or leakage on fluorescein angiography in the inferotemporal region (right eye). **G**–**H** Previous argon laser treatment in the inferotemporal region (left eye). **I**: Hyperfluorescent spots in the superonasal retina. **L** Hyperfluorescent spots in the superotemporal retina

DCP analysis showed telangiectasia and microaneurysms in the S, N and T quadrants in 8 eyes (80%), while I quadrant was involved in 7 eyes (70%). Vessels tortuosity was similar, with 6 eyes (60%) affected in S, 5 eyes (50%) in the N, 7 patients (70%) in I, and 8 eyes (80%) in the T quadrant. Increased ischemia was observed in the S quadrant in 4 eyes (40%), in the N quadrant in 3 eyes (30%), in the I quadrant in 7 eyes (70%), and in the T quadrant in 8 eyes (80%). Intraretinal cysts were more prevalent, seen in 9 patients (90%) in the S quadrant, in 8 eyes (80%) in the N quadrant; in 6 patients (60%) in the I quadrant, and in 8 patients (80%) in the T quadrant. At the equatorial level FA revealed leakage in 20% of cases and vascular hyperfluorescence in 60% (Table 2). Only two patients (20%) had undergone Argon laser treatment in the ischemic equatorial retina before arriving at our hospital. The quadrants involved were eT in 40% of cases, eI-eT in 40%, eS-eN in 10%, and eS-eT in 10%. According to the aforementioned data, 80% of patients with MacTel type 1 exhibited equatorial retinal involvement due to either vascular hyperfluorescence or leakage in the equatorial retina (Fig. 4).

**Table 2:** Percentage of patients with retinal vascular alterations in each capillary plexus

	SCP*				DCP **			
	Temporal (%)	Inferior (%)	Superior (%)	Nasal (%)	Temporal (%)	Inferior (%)	Superior (%)	Nasal (%)
Teleangectasia & microaneurysm	80	70	60	60	80	70	80	80
Tortuosity	80	70	50	30	80	70	60	50
Ischemia	70	80	60	50	80	70	40	30
Intraretinal cysts	40	20	40	30	80	60	90	80
Leakage	0	0	0	0	70	0	0	0

* SCP Superficial capillary plexus, **DPC Deep capillary plexus

## Discussion

This report presents a study on a cohort diagnosed with MactTel Type 1 focusing on clinical characteristics, multimodal imaging findings, and treatment responses with a long follow up. All cases were unilateral, predominantly affecting males, as reported by Gass et al. [1, 2, 11]. OCT-A analysis revealed teleangectasias and microaneurysms primarily in the T (80%) and I (70%) quadrants across both the SCP and DCP, with involvement also noted in the superior and nasal quadrants of the DCP. Vessel tortuosity was observed primarily in the T (80%) and I (70%) sides for both SCP and DCP, with ischemic alterations in the T (70%) and I (80%) sides for SCP, and in the T (80%) and I (70%) side for DCP.

Several studies have documented the involvement of the temporal quadrants in MacTel Type 1, with Cirafici et al. specifically noting a mild reduction in SCP capillary density and numerous deep-layer telangiectasias via OCT-A [5] Edema associated to intraretinal cysts, primarily located in the T (40%) and S quadrant (40%) of SCP, and more extensively in the DCP (temporal 80%, superior 90%, and nasal 80%), along with 70% T quadrant involvement revealed by FA, mirroring a common finding in various investigations. In particular, Abujamra et al. reported late dye leakage in the S, T, and I quadrants, as well as in the SN and IN areas in 68% of cases [12]. Currently, there is no standardized treatment available for MacTel type (1) Some authors suggest that anti-VEGF therapy may show slight efficacy in MacTel type 2 compared to type 1, although no consensus has been reached on this matter [8, 13]. Indeed, research by Takayama et al. and Moon et al. on the response to Bevacizumab suggests that anti-VEGF therapy is less effective in MacTel Type 1 compared to Type (2) This limited efficacy is attributed to the distinct pathophysiology of Type 1, which lacks neovascularization. Consequently, all patients continued to exhibit intraretinal fluid even after undergoing treatment [8, 13].

In our study, the effectiveness of Anti-VEGF therapy was generally minimal, with only two patients experiencing minor response following Ranibizumab injections. Variability in anti-VEGF across studies may be due to differences in VEGF-A expression. This variation could be influences by higher levels of placental growth factor in MactTel Type 1 patients, potentially improving their response to Aflibercept [15]. Conversely, the present study confirmed a favorable response to DEX treatment.

Cirafici et al. and Loutfi et al. observed a strong response to steroid therapy similar to our findings [3, 5]. The efficacy of intravitreal long-acting steroids in reducing macular edema and exudation in MactTel Type 1 can be attributed to the suppression of inflammatory cytokines, as well as the anti-inflammatory properties of DEX, which aid in stabilizing the blood retinal barrier. Loutfi et al. compared DEX with Triamcinolone noting that while Triamcinolone effectively treated macular edema, it caused more side effects like cataracts and increased intraocular pressure, limiting its use [3]. Conversely, the DEX showed a safer profile, resulting in favorable anatomical outcomes with fewer injections and a lower risk of endophthalmitis, compared to anti-VEGF therapies [3]. In 2006, Cakir et al. reported that three patients treated with a single triamcinolone injection resulted in reduced macular edema and retinal thickness at multimodal imaging, confirming its utility in various pathologies such as diabetic macular edema, uveitis with cystoid macular edema associated and idiopathic juxtafoveal teleangectasias [14]. Corticosteroids, by stabilization of blood retinal barrier permeability, have also been used to reduce intraocular inflammation or vascular leakage. Following anti-VEGF treatment failures, seven MacTel Type 1 cases treated with DEX implants as a second-line therapy showed substantial yet temporary reductions in macular edema and exudation, with manageable side effects [6, 17, 18]. As a matter of fact, none of our patients experienced cataract development or increased intraocular pressure, and the anatomical and functional benefits were evident, although transient. Additionally, laser photocoagulation has proven effective in treating macular telangiectasias, especially when applied in a grid pattern to the temporal side or the posterior pole [6, 19, 20]. Consistently, according to our experience, some patients pre-treated with argon laser for capillary leakage in the equatorial retina showed significant improvements. The combined use of argon laser to treat peripheral retinal leakage and ischemia, and a dexamethasone implant to manage inflammation in the peripheral retina and macular edema, may represent a viable therapeutic alternative for patients presenting with these specific characteristics [6, 18, 19, 19, 20].

According to the current data, 60% of patients exhibited vascular hyperfluorescence, while 20% presented leakage. Additionally, 20% of patients had undergone argon laser treatment for leakage at the time of diagnosis, specifically in the equatorial retina on FA. The quadrants mainly involved were eT and eI-eT both in 40% of cases, while eS-eN and eS-eT were involved in 10% of cases. Among them, the patients received an initial treatment of 6 intravitreal injections of Ranibizumab, 1 of Aflibercept, and 3 of DEX. Among the 6 patients treated with Ranibizumab, 2 had equatorial leakage and 4 had equatorial vascular hyperfluorescence. After the first cycle, there was a total reduction of leakage in both cases, while among the 4 patients with vascular hyperfluorescence, there was a total reduction in 2 cases and a partial reduction in the other 2. The patient treated with Aflibercept had already undergone laser treatment in the equatorial retina. Among the 3 patients treated with DEX, 1 had already been treated with laser, and 2 had vascular hyperfluorescence that partially decreased after the injection.

## Conclusions

This study presents a comprehensive evaluation of idiopathic MacTel Type 1, delineating its clinical profile, imaging characteristics, and therapeutic responses over a mean follow up of 3 years. We found that the majority of the patients with MacTel Type 1 exhibited vascular hyperfluorescence or leakage in the equatorial retina, with greater involvement of the inferotemporal region. OCT-A particularly highlights the reduction in capillary density and the prominence of vascular anomalies. In line with previous reports, our data further substantiate the limited utility of anti-VEGF therapy for MacTel Type 1, reinforcing the concept that its etiology may not be primarily be driven by VEGF. Conversely, the positive anatomical outcomes observed with DEX implants in our cohort add to the cumulative evidence supporting its use as a more effective treatment modality. However, future studies with larger patient cohorts and longer follow-up are warranted to validate these findings and establish more robust, statistically significant evidence.
